# Tyrosine Kinase Inhibitors, Antibody–Drug Conjugates, and Bispecific Antibodies in Oncogene-Driven Non-Small-Cell Lung Cancer: Evolving Roles in Treatment Sequencing and Resistance Management

**DOI:** 10.3390/ijms27146251

**Published:** 2026-07-14

**Authors:** Saba Musleh Ud Din, Amy Kiamos, Sundas Ali, Meri Muminovic Mehta, Luis E. Raez

**Affiliations:** Department of Hematology and Oncology, Memorial Healthcare System, 703 North Flamingo Road, Pembroke Pines, FL 33028, USA

**Keywords:** non-small-cell lung cancer, tyrosine kinase inhibitors, antibody–drug conjugates, bispecific antibodies, targeted therapy, resistance mechanisms, treatment sequencing, precision oncology

## Abstract

The treatment landscape of oncogene-driven non-small-cell lung cancer (NSCLC) has evolved substantially with the development of targeted therapies directed against actionable molecular alterations. Tyrosine kinase inhibitors (TKIs) remain the cornerstone of treatment for many driver-defined subsets; however, acquired resistance, central nervous system progression, and tumor heterogeneity continue to limit long-term disease control. This review examines the mechanistic foundations, clinical evidence, resistance patterns, and emerging therapeutic roles of TKIs, antibody–drug conjugates (ADCs), and bispecific antibodies (bsAbs) in oncogene-driven NSCLC. Relevant preclinical studies, clinical trials, and recent therapeutic advances across major actionable driver alterations were reviewed and compared. TKIs provide potent and selective inhibition of oncogenic signaling and remain the preferred frontline therapy in most molecular subgroups, whereas ADCs offer targeted payload delivery that may overcome diverse resistance mechanisms, and bsAbs provide dual-target blockade and immune-mediated antitumor activity. Emerging evidence supports the expanding role of ADCs and bsAbs in post-TKI settings and selected biomarker-defined populations. Resistance mechanisms differ across therapeutic classes and include secondary target alterations, bypass pathway activation, antigen loss, payload resistance, and receptor adaptation. Collectively, these modalities are increasingly being integrated into biomarker-guided treatment strategies, with future management likely to rely on rational sequencing and combination approaches tailored to resistance mechanisms, target expression, central nervous system involvement, and tumor heterogeneity.

## 1. Introduction

Lung cancer remains the leading cause of cancer-related mortality worldwide, with non-small-cell lung cancer (NSCLC) accounting for approximately 85% of all cases [1]. Over the past two decades, the identification of actionable oncogenic driver alterations has fundamentally reshaped the therapeutic landscape of advanced NSCLC. Approximately 50% of patients with non-squamous NSCLC harbor gene alterations now classified as actionable oncogenic drivers, a proportion that continues to increase with improved detection methods and the discovery of new targetable alterations [2]. The most prevalent of these include mutations in *EGFR* (12–48%, varying by ethnicity), *KRAS* (including G12C, ~10–31%), and gene fusions involving *ALK* (~2–5%), *ROS1* (~1–2%), and *RET* (~1–2%), as well as less common but clinically significant alterations in *MET* exon 14 skipping (~2–3%), *ERBB2/HER2* mutations (~2–3%), *BRAF* V600E (~1–2%), *NTRK1/2/3* fusions (1%), and *NRG1* fusions (1%) [3,4]. Substantial differences exist between populations, with Asian cohorts exhibiting higher rates of *EGFR* mutations and lower rates of *KRAS* G12C mutations compared with Western populations [2]. Current guidelines from the National Comprehensive Cancer Network (NCCN) and the American Society of Clinical Oncology (ASCO) recommend comprehensive multigene panel testing (MGPT) at diagnosis to identify all recognized biomarkers, as treatment is specific for each alteration [5,6]. Although immune checkpoint inhibitors (ICIs) have transformed the treatment of NSCLC without actionable driver alterations, their efficacy in oncogene-driven subtypes remains limited, and this review therefore focuses on TKIs, ADCs, and bispecific antibodies as the principal therapeutic modalities for this population [7].

Tyrosine kinase inhibitors (TKIs) have served as the cornerstone of targeted therapy for oncogene-driven NSCLC since the approval of first-generation EGFR inhibitors. Successive generations of TKIs have delivered progressively improved efficacy, selectivity, and central nervous system (CNS) penetration across multiple driver subtypes [2,8]. However, despite remarkable initial responses, virtually all patients treated with TKIs eventually develop resistance, whether through on-target kinase domain mutations, bypass signaling activation, or histologic transformation [9,10]. The median overall survival for patients receiving first-line EGFR-TKI monotherapy remains approximately three years, with estimated real-world five-year survival below 20% [11]. Resistance to third-generation EGFR-TKIs such as osimertinib is diverse and polyclonal, with the most common identifiable mechanisms being secondary EGFR pathway alterations (e.g., C797S) and MET pathway activation, though up to 50% of patients lack an identified resistance mechanism [11,12]. Similarly, in ALK-rearranged NSCLC, resistance to second-generation inhibitors frequently involves the G1202R solvent-front mutation, which can be overcome by the third-generation inhibitor lorlatinib, though compound mutations may subsequently emerge [5,9]. These patterns of inevitable resistance, CNS progression, and tumor heterogeneity represent critical unmet needs that have catalyzed the development of novel therapeutic modalities [10].

Into this landscape, two competing therapeutic classes have rapidly emerged. Antibody–drug conjugates (ADCs) represent a class of cytotoxic precision agents that combine the targeting specificity of monoclonal antibodies with the cell-killing potency of chemotherapeutic payloads, offering activity that is often independent of the specific resistance mutation driving TKI failure [2,13]. Bispecific antibodies (bsAbs), exemplified by the EGFR-MET bispecific amivantamab, represent dual-targeting and immune-engaging biologics capable of simultaneously blocking multiple receptor pathways and recruiting immune effector cells [12]. Both classes have achieved regulatory approvals in NSCLC and are now being evaluated across multiple lines of therapy and oncogene-driven subtypes.

The central question confronting the field is as follows: Where do TKIs, ADCs, and bispecific antibodies each optimally fit within modern treatment algorithms, and will one class ultimately dominate? This review examines the mechanistic foundations, comparative molecular strengths and weaknesses, and emerging clinical positioning of these three therapeutic classes in oncogene-driven NSCLC.

## 2. Mechanistic Foundations of Each Therapeutic Class

### 2.1. Tyrosine Kinase Inhibitors (TKIs)

TKIs are small-molecule inhibitors that block the catalytic activity of receptor tyrosine kinases by competing with adenosine triphosphate (ATP) at the kinase domain binding site or, less commonly, by engaging allosteric regulatory regions [2,8]. Their pharmacologic properties—oral bioavailability, relatively small molecular size, and the capacity for rational structural optimization—have enabled the development of multiple generations of inhibitors with progressively improved potency, selectivity, and CNS penetration profiles.

In EGFR-mutant NSCLC, first-generation reversible inhibitors (erlotinib, gefitinib) were supplanted by the second-generation irreversible inhibitor afatinib and subsequently by the third-generation mutant-selective inhibitor osimertinib, which covalently binds the C797 residue and retains activity against the T790M resistance mutation [14]. Osimertinib demonstrates superior CNS penetration compared with earlier-generation agents and is the current standard first-line therapy [15]. Lazertinib, another third-generation EGFR-TKI with high CNS penetrance, has been approved in combination with amivantamab [11]. In ALK-rearranged NSCLC, the therapeutic armamentarium spans from the first-generation inhibitor crizotinib through second-generation agents (alectinib, brigatinib, ceritinib, ensartinib) to the third-generation inhibitor lorlatinib, each offering broader resistance mutation coverage and improved intracranial activity [16]. Lorlatinib provides the broadest resistance mutation coverage, including activity against the G1202R mutation and compound mutations, with an intracranial complete response rate of 71% in the CROWN trial [5,16]. For other driver subtypes, selective TKIs include capmatinib and tepotinib (MET exon 14 skipping), selpercatinib and pralsetinib (RET fusions), crizotinib, entrectinib, repotrectinib, and taletrectinib (ROS1 fusions), dabrafenib/trametinib and encorafenib/binimetinib (BRAF V600E), sotorasib and adagrasib (KRAS G12C), and the recently approved zongertinib and sevabertinib (HER2 mutations) [15].

The dominant mechanisms of resistance to TKIs can be broadly categorized as on-target and off-target [9,17]. On-target resistance involves secondary mutations within the kinase domain that impair drug binding_,_ exemplified by EGFR T790M (resistance to first/second-generation TKIs), EGFR C797S (resistance to osimertinib), and ALK G1202R (resistance to second-generation ALK inhibitors) [10,14,18,19]. Additional on-target EGFR resistance mutations identified after osimertinib include L718Q, L792H, and G796/C797 alterations [19]. Off-target resistance mechanisms include bypass signaling activation (most commonly MET amplification, HER2/HER3 amplification, and oncogenic fusions), downstream pathway reactivation (RAS/MAPK, PI3K/AKT), cell-cycle gene alterations, and histologic transformation to small cell lung cancer or squamous cell carcinoma [9,17]. Notably, resistance is frequently polyclonal, with multiple mechanisms coexisting within the same patient, and up to 50% of patients progressing on osimertinib lack an identifiable molecular resistance mechanism [11,12].

### 2.2. Antibody–Drug Conjugates (ADCs)

ADCs are engineered biopharmaceuticals composed of three structural components: a monoclonal antibody directed against a tumor-associated surface antigen, a chemical linker, and a cytotoxic payload [20,21,22]. The antibody confers target specificity and facilitates receptor-mediated endocytosis upon binding to the cell-surface antigen. Following internalization, the ADC is trafficked through the endosomal-lysosomal pathway, where the linker is cleaved (in the case of cleavable linkers) or the antibody is degraded (for non-cleavable linkers), releasing the cytotoxic payload intracellularly [23,24].

Linker chemistry is a critical determinant of ADC pharmacology. Cleavable linkers—including acid-labile hydrazones, protease-sensitive peptide sequences (e.g., the valine-citrulline dipeptide or the glycine-glycine-phenylalanine-glycine tetrapeptide used in deruxtecan-based ADCs), and disulfide bonds—are designed to release the payload selectively within the tumor microenvironment or upon lysosomal processing [20,25]. Non-cleavable linkers, such as the thioether linker in ado-trastuzumab emtansine (T-DM1), require complete antibody degradation for payload release and generally produce less membrane-permeable metabolites, limiting the bystander effect [22,26]. The drug-to-antibody ratio (DAR) influences both efficacy and tolerability; trastuzumab deruxtecan (T-DXd) achieves a high DAR of approximately 8, contributing to its potent antitumor activity [27,28].

The cytotoxic payloads employed in NSCLC-relevant ADCs fall into two principal categories. Topoisomerase I inhibitors, exemplified by the deruxtecan (DXd) payload used in trastuzumab deruxtecan and datopotamab deruxtecan, induce DNA single-strand breaks during replication, leading to replication fork collapse and apoptosis [27,29]. Microtubule-disrupting agents, including the monomethyl auristatin E (MMAE) payload in telisotuzumab vedotin and the maytansinoid in ado-trastuzumab emtansine, inhibit tubulin polymerization and arrest cells in mitosis [20,21].

A pharmacologically distinctive feature of certain ADCs is the bystander effect—the capacity of released membrane-permeable payloads to diffuse into and kill neighboring tumor cells that may not express the target antigen [26,30]. This property is particularly relevant in the context of tumor heterogeneity, where antigen expression may be variable across the tumor mass. ADCs with cleavable linkers and hydrophobic, membrane-permeable payloads (e.g., T-DXd) demonstrate a robust bystander effect, which may partly explain their efficacy in tumors with heterogeneous or low-level antigen expression [30,31]. In contrast, ADCs with non-cleavable linkers (e.g., T-DM1) generate charged, membrane-impermeable metabolites with minimal bystander killing, which may limit their efficacy in antigen-heterogeneous tumors [26,30].

ADCs currently approved or in advanced clinical development for NSCLC include trastuzumab deruxtecan (anti-HER2; approved for HER2-mutant NSCLC and HER2 IHC 3+ tumors); datopotamab deruxtecan (anti-TROP2; approved for EGFR-mutant NSCLC after prior EGFR-directed therapy and platinum-based chemotherapy); and telisotuzumab vedotin (anti-c-MET; approved for c-MET-high non-squamous NSCLC after prior systemic therapy) [27]. The toxicity profile of ADCs is shaped by the target antigen, the cytotoxic payload, and the linker chemistry. Linker design is a critical determinant of ADC safety: cleavable linkers may release unpredictable amounts of payload extracellularly within the proteolytic-rich tumor microenvironment, enabling a bystander effect but also allowing free payload to re-enter the bloodstream and cause off-target systemic toxicities [32,33]. A large-scale pharmacovigilance analysis of FDA-approved ADCs demonstrated distinct component-specific toxicity signatures: ADCs with cleavable linkers showed enrichment for pulmonary toxicity and infections, while non-cleavable linker ADCs exhibited a distinctive pattern dominated by ocular toxicity, particularly corneal epithelial and stromal injury [34]. Furthermore, a comprehensive analysis of over 7800 patients across 40 clinical trials found that ADCs with cleavable linkers had a 47% severe toxicity rate compared to 34% for non-cleavable linkers, reflecting the trade-off between enhanced bystander-mediated antitumor activity and increased off-target effects [35]. The drug-to-antibody ratio (DAR) further modulates this relationship, with DAR > 5 ADCs demonstrating heterogeneous multi-organ toxicity profiles spanning metabolic, hepatic, pulmonary, and neurological domains [36].

Interstitial lung disease (ILD) is the most clinically significant toxicity, occurring in approximately 10–15% of patients treated with T-DXd, with rare fatal cases [28,37]. Importantly, ILD/pneumonitis is not exclusive to T-DXd but has been reported across multiple ADC classes, including datopotamab deruxtecan (8.8% in TROPION-Lung01), sacituzumab govitecan, and other deruxtecan-based and non-deruxtecan ADCs, though incidence rates and severity vary substantially by payload, linker chemistry, and DAR [34,38]. A real-world pharmacovigilance analysis confirmed that even among topoisomerase I inhibitor-based ADCs sharing the same payload class, divergent pulmonary toxicity profiles emerge: T-DXd demonstrated a strong ILD signal (ROR 34.6), whereas sacituzumab govitecan did not exhibit a significant ILD signal, likely reflecting differences in linker chemistry and DAR [38]. These findings underscore that ILD risk assessment should be individualized to each ADC rather than generalized across the class.

Other class-relevant toxicities include gastrointestinal effects (nausea, mucositis), hematologic cytopenias, and ocular toxicity, which vary by payload and target [24,39].

Unlike TKIs, ADC efficacy does not depend on inhibition of a specific signaling pathway; rather, their cytotoxic activity is mediated by the payload, which allows them to retain activity in the setting of diverse TKI resistance mechanisms, including on-target kinase mutations and bypass signaling activation [40,41]. However, their effectiveness is influenced by target antigen expression and internalization efficiency, with preclinical data demonstrating a strong linear relationship between receptor density and intracellular payload exposure [42,43]. Notably, this relationship is not absolute: ADCs with potent bystander effects, such as trastuzumab deruxtecan, have demonstrated efficacy even in tumors with heterogeneous or low-level antigen expression, as the membrane-permeable payload can diffuse into neighboring antigen-negative cells [30,44]. In addition, the larger molecular size of ADCs (~150 kDa) limits blood–brain barrier penetration relative to small-molecule TKIs. Nevertheless, emerging clinical evidence demonstrates intracranial activity for several ADCs in NSCLC, including trastuzumab deruxtecan in ERBB2-mutant disease, datopotamab deruxtecan (intracranial ORR 22% in patients with measurable brain lesions), and patritumab deruxtecan in patients with active brain metastases (TUXEDO-3) [45,46]. Proposed mechanisms facilitating CNS activity include disruption of the blood–brain barrier at metastatic sites (replaced by a blood-tumor barrier with higher endothelial fenestration), membrane-permeable payloads, high drug-to-antibody ratios, and bystander effects [45,47]. Toxicity profiles are driven largely by the payload, with ILD/pneumonitis representing the most clinically significant concern. A meta-analysis reported an overall all-grade ILD incidence of 5.86% across ADCs, rising to 13.58% with trastuzumab deruxtecan specifically [48]. In NSCLC, ILD rates are the highest among solid tumors, reaching 26% at the 6.4 mg/kg dose in DESTINY-Lung01 and 14.9% at the approved 5.4 mg/kg dose in DESTINY-Lung02, with rare fatal cases [27,49]. ILD incidence does not appear to correlate with target antigen expression, suggesting that payload and linker characteristics are the primary determinants of pulmonary toxicity [50,51].

### 2.3. Bispecific Antibodies (bsAbs)

Bispecific antibodies are engineered immunoglobulin-based molecules designed to simultaneously engage two distinct epitopes or antigens [52,53]. In the context of NSCLC, the prototypical and most clinically advanced bsAb is amivantamab, a fully human IgG1-based bispecific antibody that binds the extracellular domains of both EGFR and MET [52].

The mechanism of action of amivantamab is multifaceted and distinct from that of TKIs. First, amivantamab blocks ligand binding to both EGFR and MET, inhibiting receptor activation and downstream signaling through the RAS/MAPK and PI3K/AKT pathways [54]. Second, amivantamab induces receptor internalization and lysosomal degradation, effectively downregulating surface expression of both EGFR and MET—a mechanism that is not dependent on the specific kinase domain mutation and can therefore bypass TKI resistance mutations such as C797S and exon 20 insertions [52,53,55]. Third, amivantamab engages immune effector cells through its low-fucose, optimized Fc domain, mediating antibody-dependent cellular cytotoxicity (ADCC) via natural killer cells and antibody-dependent cellular trogocytosis (ADCT) via macrophages [56,57]. Preclinical studies have demonstrated that MET expression on tumor cells enhances amivantamab binding to EGFR and augments ADCC activity, providing a mechanistic rationale for the dual-targeting approach [58].

The structural format of amivantamab is an IgG1-like bispecific with one arm binding EGFR domain III and the other binding the MET Sema domain [52]. Other bispecific formats under investigation in NSCLC include dual-Fab constructs and Fc-engineered molecules designed to optimize valency, half-life, or effector function. Beyond amivantamab, zenocutuzumab is a bispecific antibody targeting HER2 and HER3 that has been approved for NRG1 fusion-positive NSCLC; it functions by blocking neuregulin-1-mediated HER2–HER3 heterodimerization and downstream signaling [59]. Multi-target bsAbs engaging other receptor combinations (e.g., EGFR/HER2, ALK/MET) are in preclinical and early clinical development [60].

**Figure 1:**
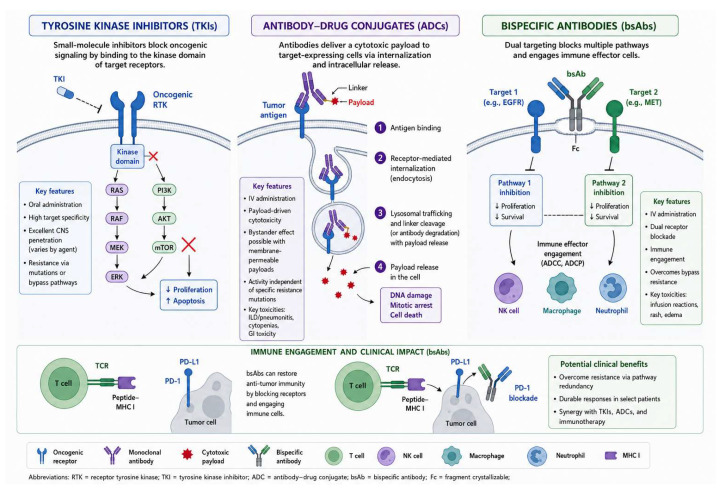
Mechanistic overview of TKIs, ADCs, and bispecific antibodies in oncogene-driven NSCLC. TKIs (**left**) block intracellular kinase domain signaling through RAS/MAPK and PI3K/AKT/mTOR pathways. ADCs (**center**) deliver cytotoxic payloads via antigen-mediated internalization and lysosomal release, inducing DNA damage or mitotic arrest. Bispecific antibodies (**right**) simultaneously engage two receptor targets (e.g., EGFR and MET), inhibiting dual signaling pathways and recruiting immune effector cells via ADCC and ADCP. Bottom panel illustrates bsAb-mediated immune engagement and potential restoration of antitumor immunity. Solid arrows indicate signaling pathways or biologic processes, dashed arrows represent indirect or sequential processes, and red cross symbols indicate inhibition or blockade. Colors are used to distinguish different therapeutic mechanisms and signaling pathways.

The toxicity profile of bispecific antibodies differs from that of both TKIs and ADCs. Infusion-related reactions are the most common acute toxicity, occurring predominantly during early treatment cycles and manageable with premedication and dose modifications [53]. Dermatologic toxicity (acneiform rash, paronychia) and peripheral edema reflect on-target EGFR and MET pathway modulation, respectively [53]. Venous thromboembolism has been observed at increased rates in combination regimens [11,12]. The requirement for intravenous administration (though subcutaneous formulations are in development) represents a logistical consideration compared with oral TKIs. The distinct mechanisms of action of TKIs, ADCs, and bispecific antibodies are summarized in Figure 1.

### 2.4. Comparative Molecular Strengths and Weaknesses

A comparative overview of the major molecular characteristics, strengths, and limitations of each therapeutic class is provided in Table 1. Each therapeutic class possesses distinct molecular advantages and limitations that inform their positioning within treatment algorithms.

#### 2.4.1. TKI

TKIs offer unparalleled molecular precision for defined kinase domain targets, with high oral bioavailability and, for later-generation agents, excellent CNS penetration (intracranial ORR up to 71–87% for lorlatinib and alectinib) [5,16]. Their principal vulnerability is susceptibility to single-point resistance mutations that alter the drug-binding site, as well as bypass signaling activation that renders continued kinase inhibition insufficient [9,17]. The oral route of administration and generally manageable toxicity profile support long-term use and patient convenience.

#### 2.4.2. ADC

ADCs derive their potency from the cytotoxic payload rather than from inhibition of a specific signaling pathway, rendering them effective against tumors harboring diverse resistance mutations, provided the target antigen is expressed on the cell surface [2,13]. The bystander effect extends their activity to antigen-heterogeneous tumors [30]. However, ADC efficacy is fundamentally dependent on adequate antigen expression and efficient internalization, and their large molecular size limits blood–brain barrier penetration, though emerging data suggest intracranial activity for newer ADCs such as T-DXd and datopotamab deruxtecan, likely facilitated by BBB disruption at metastatic sites and membrane-permeable payloads [16,47]. ILD/pneumonitis remains a significant safety concern [37].

#### 2.4.3. Bispecific Antibodies

Bispecific antibodies uniquely combine simultaneous blockade of two receptor pathways with immune effector cell engagement, offering a mechanistic approach to bypass pathway-mediated resistance (e.g., MET amplification after EGFR-TKI) without relying on cytotoxic payloads [12]. Receptor downregulation via internalization and degradation provides an additional mechanism independent of kinase domain mutations [52,53]. However, bsAbs share the CNS penetration limitations of large-molecule biologics, require intravenous administration, and their immune-mediated mechanisms may be influenced by the tumor immune microenvironment [47,57]. Infusion reactions and the logistical burden of IV therapy are practical considerations [53].

## 3. Clinical Evidence Across Therapeutic Classes

### 3.1. Tyrosine Kinase Inhibitors (TKIs): The Initial Backbone

#### 3.1.1. EGFR-TKI

The treatment of oncogene-driven NSCLC has evolved substantially after the introduction of targeted TKIs over the past two decades. Epidermal growth factor receptor (EGFR) gene mutations in NSCLC are one of the most common actionable mutations, leading to the development of three generations of EGFR-TKIs encompassing five drugs being approved for the treatment of EGFR-mutated NSCLC [61]. Osimertinib, a third-generation EGFR-TKI, became a first-line standard of care for advanced-stage or metastatic EGFR-mutated (exon 19 deletion or L858R mutation) NSCLC after significantly improving progression-free survival (PFS) to 18.9 months compared to 10.2 months with earlier-generation EGFR-TKIs (HR 0.46; 95% CI 0.37–0.57; *p* < 0.001), with fewer grade 3 toxicities and higher central nervous system (CNS) activity in the phase III FLAURA trial [62]. In the follow-up phase III FLAURA2 trial, the addition of chemotherapy to osimertinib significantly improved median overall survival (OS) to 47.5 months compared to 37.6 months with osimertinib monotherapy (HR 0.77; 95% CI 0.61–0.96; *p* = 0.02), albeit with increased toxicity, fortifying the osimertinib plus chemotherapy regimen as a first-line standard for advanced-stage EGFR-mutated NSCLC [63].

#### 3.1.2. ALK-TKI

Anaplastic lymphoma kinase (ALK) gene rearrangements are another common actionable target in advanced-stage or metastatic NSCLC, leading to the development of three generations of ALK-TKIs, with six drugs being approved for ALK-positive NSCLC [64]. Alectinib, a second-generation ALK-TKI, demonstrated improved PFS of 48.2 months compared to 23.3 months with crizotinib (HR 0.43; 95% CI 0.32–0.58; *p* < 0.0001) and notably decreased rates of CNS progression (12% vs. 45%) (HR 0.16; 95% CI 76–88.5; *p* < 0.001) in advanced-stage ALK-positive NSCLC in the phase III ALEX trial [65,66]. Brigatinib, another second-generation ALK-TKI, also proved to be significantly superior compared to crizotinib with improved PFS of 67% vs. 43% (HR 0.49; 95% CI 0.33–0.74; *p* < 0.001) and compelling intracranial response rates of 78% vs. 29% in the phase III ALTA-1L trial [67]. Most impressively, in the phase III CROWN trial, lorlatinib, a third-generation ALK-TKI, did not reach median PFS at 5-year follow-up versus 9.1 months with crizotinib (HR 0.19; 95% CI 0.13–0.27) and demonstrated an improved 5-year PFS of 60% versus 8% with crizotinib (HR 0.19; 95% CI 51–68), which is notably the longest PFS documented with monotherapy targeted treatment in advanced-stage NSCLC and all metastatic solid tumors [68]. Lorlatinib had excellent intracranial response rates for brain metastases compared to crizotinib (82% vs. 23%), including 71% of patients with a complete intracranial response [69]. Lorlatinib established itself as an effective first-line standard of care for ALK-rearranged NSCLC given its robust response rates.

#### 3.1.3. KRAS G12C TKI

KRAS is the most commonly mutated oncogene in non-squamous NSCLC in Western populations, occurring in approximately 25–33% of lung adenocarcinomas. KRAS G12C is the most prevalent KRAS variant, accounting for ~40% of KRAS mutations and ~10–13% of advanced non-squamous NSCLC [70]. In previously treated advanced-stage or metastatic KRAS-mutated NSCLC patients, sotorasib demonstrated improved PFS of 5.6 months compared to 4.5 months with docetaxel (HR 0.66; 95% CI 0.51–0.86, *p* = 0.0017) with improved tolerability and fewer grade 3 toxicities in the phase III Code Break 200 trial [71]. Adagrasib demonstrated improved PFS of 5.5 months versus 3.8 months with docetaxel (HR 0.58; 95% CI 0.45–0.76; *p* < 0.0001) in previously treated patients in the phase III KRYSTAL-12 trial [72]. There are several ongoing studies evaluating use of KRAS G12C TKIs in first-line management. The phase II KRYSTAL-7 trial showed the adagrasib plus pembrolizumab combination had a median PFS of 27.7 months with tolerable toxicity in patients with KRAS G12C-mutated NSCLC with a PD-L1 ≥ 50%, ultimately leading to further evaluation with an ongoing phase III trial [73]. Further follow-up is necessary to determine the optimal frontline therapy for KRAS G12C advanced-stage NSCLC.

#### 3.1.4. HER2-TKI

There are several human epidermal growth factor receptor 2 (HER2)-targeted TKIs including pan-HER TKIs (afatinib, dacomitinib, neratinib, poziotinib, pyrotinib, tarloxotinib, mobecertinib), EGFR/HER2 TKIs (lapatinib), and HER2-specific TKIs (zongertinib, sevabertinib) [74]. In HER2-altered advanced-stage or metastatic NSCLC, the pan-HER2 TKIs were shown to have lower response rates; however, HER2-specific TKIs have proven to be efficacious [74]. In the phase I Beamion LUNG-1 trial, zongertinib (HER2-specific TKI) showed confirmed objective response in 71% (95% CI 60–80; *p* < 0.001), median PFS of 12.4 months, and no cases of interstitial lung disease (ILD) in previously treated HER2-mutant NSCLC [75]. The impressive results of this study led to accelerated FDA approval of zongertinib in 2025 and its listing as a first-line treatment option in NCCN guidelines for HER2 (ERBB2)-mutated advanced-stage NSCLC; however, phase III trials are still ongoing [75].

#### 3.1.5. MET-TKI

MET exon 14 skipping mutations (METex14) are less commonly present in advanced-stage NSCLC, although it can be associated with MET amplification, which is a known resistance mechanism to EGFR-mutated NSCLC [76]. Capmatinib and tepotinib are selective MET-TKIs that have shown to be effective for first-line therapy in advanced-stage METex14-driven NSCLC. Tepotinib demonstrated an ORR of 51.4% (95% CI 45.8–57.1) in the overall population with advanced-stage METex14-driven NSCLC and showed a higher ORR of 57.3% (95% CI 49.4–65) in treatment-naïve patients in the phase II VISION trial [77]. Capmatinib had an ORR of 68% in previously treated patients (95% CI 55–79.7) and ORR of 44% in treatment-naïve patients (95% CI 34.1–54.3) in advanced-stage METex14-driven NSCLC in the phase II GEOMETRY mono-1 trial [77].

#### 3.1.6. ROS, RET, and NTRK-TKI

ROS1, RET, and NTRK gene fusions are less common in advanced-stage NSCLC, although multiple selective TKIs including entrectinib (ROS1, NTRK), crizotinib (ROS1), selpercatinib (RET), pralsetinib (RET), and larotrectinib (NTRK) have shown high and durable response rates in frontline therapy [69,78,79,80,81,82]. Unfortunately, acquired resistance occurs across these subsets, highlighting the importance of further studies for subsequent lines of therapies.

### 3.2. Antibody–Drug Conjugates (ADCs): Quick Rise and Broad Activity

#### 3.2.1. HER2-Directed ADC

HER2 is an ERBB family transmembrane tyrosine kinase receptor that is now an approved target for ADCs in patients with advanced-stage NSCLC whose disease has progressed on prior lines of therapy. In NSCLC, HER2 (ERBB2)-activating oncogenic alterations involve mutations (commonly exon 20 mutation), amplifications, and protein overexpression that leads to uncontrolled cell growth [74]. Trastuzumab deruxtecan (T-DXd), a HER2-directed ADC with a topoisomerase I inhibitor payload, has emerged as a promising and effective therapy for HER2-mutated NSCLC [27]. In the phase II trial DESTINY-Lung01, T-DXd 6.4 mg/kg every 3 weeks demonstrated confirmed objective response in 55% of patients with a median PFS of 8.2 months (95% CI 6–11.9) and OS of 17.8 months (95% CI 13.8–22.1) [83]. Grade 3 adverse events occurred in 46% of patients, with neutropenia and anemia being the most common, and adjudicated drug-related ILD occurred in 26% of patients (8.3% grade 5) [83]. It is worth noting that the HER2-mutant cohort achieved much higher overall response rates (ORR) when compared to HER2-overexpression or amplification, which only had modest activity [83]. In the phase II trial DESTINY-Lung02, T-DXd 5.4 mg/kg showed comparable ORR when compared to 6.4 mg/kg (50% vs. 56%) with a more favorable toxicity profile. Importantly, T-DXd 5.4 mg/m2 had significantly less adjudicated drug-related ILD occurring in 14.9% of patients compared to 32% with 6.4 mg/m2, supporting this as the safest dose for FDA approval in 2022 [49].

#### 3.2.2. TROP2-Directed ADC

Trophoblast cell-surface antigen 2 (TROP-2) has become a viable therapeutic target for ADCs, as it has been found to be overexpressed in advanced-stage NSCLC regardless of mutations [84]. Datopotamab deruxtecan (Dato-DXd), a TROP-2-directed ADC with a topoisomerase I inhibitor payload, demonstrated improved median PFS of 4.4 months versus 3.7 months with docetaxel (HR 0.75; 95% CI 0.62–0.91; *p* = 0.004) and improved OS of 12.9 months versus 11.8 months (HR 0.94; 95% CI 0.78–1.14; *p* = 0.530) in previously treated advanced-stage NSCLC in the phase III TROPION-Lung01 study [85]. Dato-DXd had significantly fewer grade 3 adverse treatment-related events when compared to docetaxel (25.6% vs. 42.1%), with slightly higher rates of adjudicated drug-related ILD (8.8% vs. 4.1%) [85]. While OS was not statistically significant in the overall patient population of advanced-stage NSCLC, a pooled analysis of phase II TROPION-Lung05 and phase III TROPION-Lung01 showed that Dato-DXd had notably better outcomes in the EGFR-mutant subgroup when compared to the entire patient population [85]. Dato-DXd in previously treated EGFR-mutated advanced-stage NSCLC patients showed an ORR of 43% (95% CI 34–52), median PFS 5.8 months (95% CI 5.4–8.2), and median OS 15.6 months (95% CI 13.1–19) [86]. These results ultimately positioned Dato-DXd as a second-line treatment option post EGFR-TKI-directed therapy.

#### 3.2.3. MET-Directed ADC

MET is a protooncogene that encodes a c-MET tyrosine kinase receptor that is now an approved target for ADCs in advanced-stage NSCLC. MET alterations associated with NSCLC tumor growth include MET overexpression, MET amplification, and MET mutations (exon 14 skipping mutation) [76]. MET overexpression is more common compared to MET amplification and MET mutations and confers a negative prognosis in NSCLC [87]. Telisotuzumab vedotin (Teliso-V), a c-MET-directed ADC with a monomethyl auristatin E cytotoxic payload, showed an ORR of 34.6% (95% CI 24.2–46.2), median PFS of 5.5 months (95% CI 4.1–8.3), and median OS of 14.6 months (95% CI 9.2–25.6) in advanced-stage EGFR-wild-type NSCLC with high c-MET overexpression (IHC 3+, ≥50% tumor cells) in the phase II LUMINOSITY trial [88]. This trial determined MET testing an important biomarker and ultimately lead to the first FDA approval in May 2025 for MET-directed ADC in advanced-stage NSCLC [88].

### 3.3. Bispecific Antibodies: The New Therapeutic Class

#### 3.3.1. Amivantamab: EGFR/MET Bispecific

Amivantamab is an EGFR-MET bispecific antibody that was initially engineered to overcome EGFR- and MET-mediated resistance pathways in advanced-stage or metastatic NSCLC [55]. In the CHRYSALIS phase I trial, amivantamab demonstrated an ORR of 40% (95% CI 29–52) and median PFS of 8.3 months (95% CI 6.9—not reached) in advanced-stage EGFR-exon-20-insertion-mutated NSCLC after progression on first-line platinum-based chemotherapy [89]. The results of CHRYSALIS resulted in accelerated FDA approval and introduction as the first approved therapy for EGFR-exon-20-insertion-mutated NSCLC with progression on prior chemotherapy [90]. In the subsequent phase III PAPILLON trial, amivantamab plus chemotherapy demonstrated a significantly longer PFS of 11.4 months compared to 6.7 months with standard chemotherapy (HR 0.4; 95% CI 0.3–0.53; *p* < 0.001), ultimately establishing this new regimen as first-line treatment for EGFR-exon-20-insertion-mutated NSCLC [55].

Subsequent pivotal trials explored the addition of lazertinib, a third-generation EGFR-TKI, to amivantamab for EGFR-mutated (exon 19 deletion or exon 21 L858R substitutions) advanced-stage or metastatic NSCLC [12]. In the phase III MARIPOSA trial, amivantamab plus lazertinib demonstrated a significantly longer median PFS of 23.7 months compared to 16.6 months with osimertinib monotherapy (HR 0.70; 95% CI 0.58–0.85; *p* < 0.001), and improved survival outcomes with a 3-year OS of 60% and 51% (HR 0.75; 95% CI 0.51–0.92; *p* = 0.005) in previously untreated patients, respectively [11]. MARIPOSA ultimately established amivantamab plus lazertinib as the new standard of care for frontline therapy for advanced-stage EGFR-mutated (exon 19 deletion or exon 21 L858R substitutions) NSCLC. Notably, amivantamab plus lazertinib was associated with a higher incidence of grade ≥3 adverse events than Osimertinib (75% vs. 43%), whereas treatment discontinuation due to treatment-related adverse events was similar between the two groups (10% vs. 10%) [12]. Amivantamab plus lazertinib caused higher rates of paronychia, rashes, infusion reactions, venous thromboembolic events, and transaminitis; however, enhanced prophylactic strategies including antibiotics, skin moisturizers, anticoagulation, and steroids have helped to reduce these adverse events [11].

The phase III MARIPOSA-2 trial compared amivantamab-lazertinib-chemotherapy, amivantamab-chemotherapy, or chemotherapy alone in EGFR-mutated (exon 19 deletion or exon 21 L858R substitutions) advanced-stage or metastatic NSCLC patients who had progressed on osimertinib [91]. Amivantamab-chemotherapy and amivantamab-lazertinib-chemotherapy both significantly improved PFS (6.3 months and 8.3 months, respectively) (HR 0.48 and 0.44, respectively; *p* < 0.001) and ORR (64% and 63%) compared with chemotherapy, which had a PFS of 4.2 months and an ORR of 36% [91]. Amivantamab-chemotherapy and amivantamab-lazertinib-chemotherapy showed significantly improved intracranial PFS (12.5 months and 12.8 months, respectively) compared with 8.3 months of chemotherapy. Although amivantamab-lazertinib-chemotherapy demonstrated a modestly higher PFS of 2 months compared to amivantamab-chemotherapy, the triple regimen had considerably greater toxicity with grade ≥ 3 adverse events (92% vs. 72%) and serious treatment-emergent adverse events (52% vs. 32%) [92]. This established the doublet regimen with amivantamab-chemotherapy without lazertinib as the preferred second-line treatment in this patient population due to lower toxicities and similar efficacy [92].

#### 3.3.2. HER2/HER3 Bispecific

Neuregulin 1 (NRG1) gene fusions are rare oncogenic driver alterations, occurring in ~0.2% of solid tumors, including NSCLC, that bind to human epidermal growth factor receptor 3 (HER3), generating a HER2-HER3 heterodimerization and subsequently triggering uncontrolled cell proliferation and growth [93]. Zenocutuzumab is a bispecific antibody that targets HER2 and HER3, blocking the NRG1-fusion-protein-mediated HER2-HER3 dimerization halting downstream oncogenic signaling [38,39]. In the phase II eNRGy trial, zenocutuzumab demonstrated an ORR of 29% (95% CI 20–39), ORR of 28% in previously treated patients (95% CI 19–39), median duration of response of 12.7 months (95% CI 7.4–20.4), and median PFS of 6.8 months (95% CI 5.3–7.5) in patients with NRG1 fusion-positive advanced-stage NSCLC [59]. The most common adverse events were diarrhea, fatigue, nausea, and infusion-related reactions; however, most of the adverse events were grade 1 or 2 [94]. The results of eNRGy accelerated and established zenocutuzumab as the first FDA-approved bispecific antibody for advanced-stage NRG1 fusion-positive NSCLC after prior lines of systemic therapy [59].

#### 3.3.3. Other Novel Constructs

There are several ongoing studies evaluating bispecific antibodies beyond the current approved therapies for advanced-stage NSCLC, including PD-1/VEGFR, PD-1/CTLA4, EGFR/MET, and EGFR/HER3 [94,95,96]. Further investigations are needed to determine the definitive efficacy of these agents.

### 3.4. Immune Checkpoint Inhibitors in Oncogene-Driven NSCLC: Current Limitations and Emerging Targets

While immune checkpoint inhibitors (ICIs) targeting PD-1/PD-L1 have transformed the treatment of advanced NSCLC without actionable driver alterations, their role in oncogene-driven NSCLC remains limited. The IMMUNOTARGET registry, a multicenter retrospective study of 551 patients with oncogene-driven NSCLC treated with ICI monotherapy, demonstrated notably low objective response rates across most driver subtypes: EGFR 12%, HER2 7%, RET 6%, and ALK 0%, compared with 26% for KRAS-mutant tumors [7]. Median PFS on ICI monotherapy was only 2.1 months for EGFR-mutant and 2.5 months for ALK-rearranged NSCLC [7]. These findings are consistent with the biological characteristics of oncogene-driven tumors, which typically exhibit lower tumor mutational burden (TMB), lower PD-L1 expression, higher intratumoral heterogeneity, and a suppressed immune microenvironment with fewer infiltrating M1 macrophages and CD4+ T cells compared to wild-type tumors [97,98].

Current NCCN guidelines explicitly note that EGFR exon 19 deletion or L858R mutation, and ALK, RET, or ROS1 gene fusions have been shown to be associated with less benefit from PD-1/PD-L1 inhibitors [99]. Accordingly, first-line ICI-based regimens are contraindicated or not recommended for these molecular subgroups, and patients with these alterations are directed to targeted therapy algorithms [99]. In the post-TKI setting, the role of ICIs remains uncertain. A systematic review and network meta-analysis of ICI-based strategies in EGFR-mutant NSCLC after TKI progression found that ICI monotherapy showed inadequate efficacy, while ICI plus chemotherapy showed inconsistent results across trials, with some studies (ORIENT-31, KEYNOTE-789) demonstrating PFS improvement and others (CheckMate 722) failing to reach significance [100]. The addition of anti-angiogenic therapy to ICI-chemotherapy combinations appeared more promising but at the cost of increased toxicity [2,100].

Notably, oncogene-driven subtypes more frequently associated with tobacco exposure, such as KRAS G12C and BRAF V600E mutations, may derive greater benefit from ICIs, and KRAS-mutant NSCLC in particular shows strong ICI activity that supports first-line immunotherapy for this subgroup [2,7].

Beyond PD-1/PD-L1 and CTLA-4, several next-generation immune checkpoint targets are under active investigation in NSCLC. LAG-3 (lymphocyte activation gene-3) is the most clinically advanced, with the anti-LAG-3 antibody relatlimab approved in combination with nivolumab for melanoma and now being evaluated in NSCLC [101,102]. TIM-3 (T-cell immunoglobulin and mucin-domain containing-3) promotes T-cell exhaustion through interactions with galectin-9, CEACAM1, and HMGB1; anti-TIM-3 agents including sabatolimab and cobolimab are in phase II–III trials across multiple malignancies including NSCLC [101,103]. TIGIT (T-cell immunoreceptor with immunoglobulin and ITIM domains) suppresses T-cell and NK-cell cytotoxicity through CD155 binding, and agents such as tiragolumab are being evaluated in phase 3 NSCLC trials, though initial results have been mixed [101,103]. VISTA (V-domain Ig suppressor of T-cell activation) mediates myeloid-driven T-cell suppression through a PD-1-independent mechanism and is in early phase evaluation [101,103]. B7-H3 (CD276) is overexpressed in NSCLC tumors and is being explored both as an immune checkpoint target and as an ADC target (e.g., DS-7300), representing a potential convergence of checkpoint biology and ADC technology [101,104]. Gene expression profiling of lung adenocarcinoma has revealed heterogeneous patterns of immune checkpoint overexpression, with distinct tumor subsets showing enrichment for different checkpoint combinations, suggesting that biomarker-guided selection of checkpoint targets may be necessary for optimal therapeutic benefit [104].

The limited efficacy of current ICIs in most oncogene-driven NSCLC subtypes, combined with the emerging landscape of next-generation checkpoint targets, underscores the rationale for the present review’s focus on TKIs, ADCs, and bispecific antibodies as the primary therapeutic modalities for this patient population. However, as novel checkpoint inhibitors and combination strategies mature, their integration into treatment algorithms for oncogene-driven NSCLC—particularly in the post-TKI resistance setting or in combination with targeted agents—warrants continued investigation.

## 4. Positioning of TKIs vs. ADCs vs. Bispecifics in Treatment Algorithms

### 4.1. First-Line Therapy

**Figure 2:**
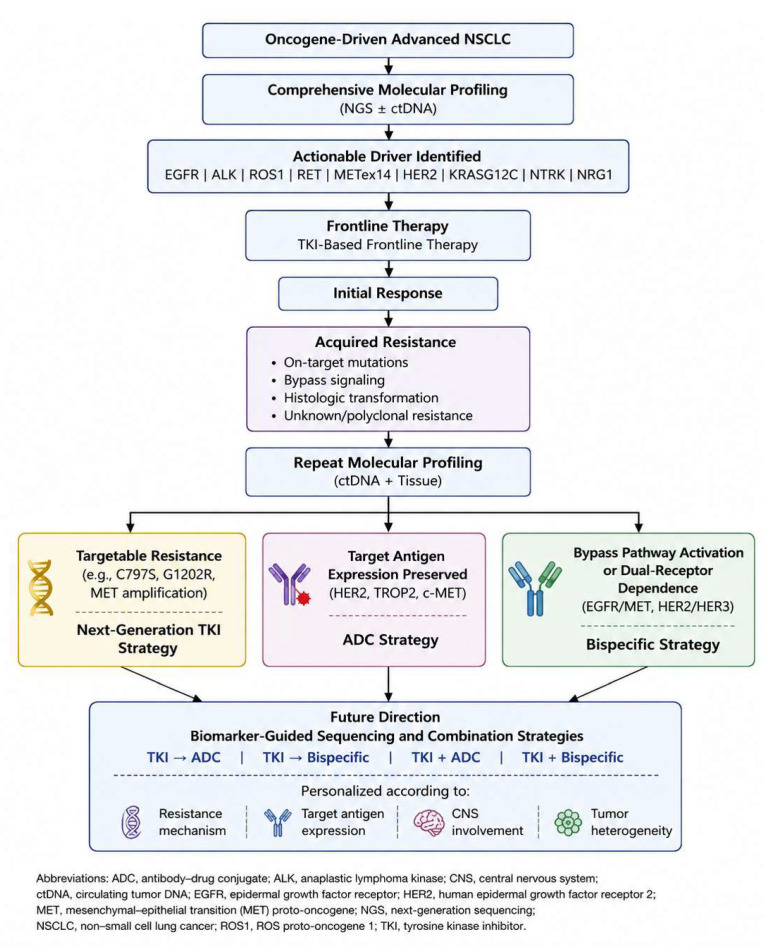
Evolving positioning of tyrosine kinase inhibitors (TKIs), antibody–drug conjugates (ADCs), and bispecific antibodies (bsAbs) in oncogene-driven advanced non-small-cell lung cancer (NSCLC). Following comprehensive molecular profiling, TKIs remain the preferred frontline therapy for most actionable driver alterations. Upon acquired resistance, repeat molecular testing using tissue and/or circulating tumor DNA (ctDNA) can identify targetable resistance mechanisms, preserved target antigen expression, or dual-pathway dependence that may guide selection of next-generation TKIs, ADCs, or bispecific antibodies, respectively. Future treatment paradigms are expected to incorporate biomarker-guided sequencing and rational combination strategies based on resistance mechanisms, target antigen expression, central nervous system involvement, and tumor heterogeneity.

TKIs remain the predominant frontline treatment for most advanced-stage or metastatic NSCLC harboring actionable oncogene-driven alterations. The evolving integration of TKIs, ADCs, and bispecific antibodies within contemporary treatment algorithms is illustrated in Figure 2. Many studies support frontline use of TKIs given their greater efficacy, favorable tolerability, convenient oral formulations, and higher intracranial response rates compared to chemotherapy [105]. Historically, HER2-mutated NSCLC first-line treatment was shaped by T-DXd, an ADC; however, this is now being challenged by zongertinib, HER2-TKI, for first-line treatment [75]. A notable exception to TKI frontline use is KRAS G12C-mutated NSCLC, although further studies are underway evaluating frontline use [106].

Treatment for metastatic NSCLC is rapidly expanding beyond TKI monotherapy, with evolving strategies to augment TKI backbones with ADCs or bispecific therapy. For example, the amivantamab-lazertinib combination improved PFS and OS compared to osimertinib monotherapy, providing clinical evidence for frontline use of TKI plus bispecific therapy to improve outcomes [11,12]. Current studies are evaluating osimertinib in combination with ADCs, including datopotamab deruxtecan (Dato-DXd) and telisotuzumab vedotin-tllv (Teliso-V), for EGFR-mutated NSCLC with the goal of overcoming resistance mechanisms [107,108]. As further phase III trials are performed and data mature, first-line therapy for advanced-stage oncogenic-driven NSCLC is likely going to shift away from TKI monotherapy to doublet or triplet regimens with TKIs, ADCs, and bispecific antibodies [108].

### 4.2. Post-TKI Progression

ADCs and bispecific antibodies have become critical in the post-TKI treatment strategy for NSCLC because they can both overcome intrinsic cellular resistance mechanisms. ADCs are effective at bypassing drug resistance mutations by delivering cytotoxic payloads directly to antigen-expressing tumor cells, ultimately bypassing downstream intracellular signaling resistance patterns [40]. Bispecific antibodies overcome TKI resistance by simultaneously blocking extracellular dual-receptors causing receptor degradation and immune-mediated cytotoxicity, also bypassing intracellular downstream signaling resistance mutations [54]. Examples include amivantamab-based combinations and Dato-DXd, both of which have proven to be efficacious for treating post-osimertinib EGFR-mutated NSCLC [85,91].

Amivantamab-based combination therapy has demonstrated robust efficacy in overcoming several osimertinib resistance mechanisms including EGFR-dependent resistance, MET-dependent resistance (MET amplification), EGFR/MET-dependent resistance, and unknown resistance patterns [109]. At the time of progression, it is essential to obtain biomarker testing to evaluate resistance mechanisms and guide next-line treatment. Selection of subsequent therapy increasingly depends on resistance profiling, target antigen expression, and pathway dependence, as summarized in Figure 2.

### 4.3. Tumor Heterogeneity and Mixed Resistance Patterns

Tumor heterogeneity represents intrinsic genomic diversity within a single tumor, which can pose challenges in next-line treatment decisions due to coexisting driver mutations, differing antigen expression, and resistance mechanisms driving oncogenesis [110]. Within a single tumor, multiple oncogenic driver alterations can accumulate, and a biopsy of only a portion of a tumor may not represent the full genomic diversity [110]. ADCs have shown effectiveness in tumor heterogeneity because the bystander effect allows for cytotoxic payloads to be delivered to adjacent cells regardless of their antigen expression [30]. Bispecific antibodies are able to overcome tumor heterogeneity by engaging dual-extracellular receptors concurrently to target two separate oncogenic intracellular signaling pathways to overcome mutations and bypass pathways; however, this typically requires surface receptor expression on tumor cells [111]. Rechallenging with TKIs also remains a viable strategy that can be used to overcome tumor heterogeneity using the available guidance of circulating tumor DNA (ctDNA) analysis for molecular responses to overcome resistance patterns [112].

### 4.4. Central Nervous System (CNS) Disease Management

Brain metastases can occur in up to 50% of patients with advanced-stage NSCLC, with about 20% occurring at initial diagnosis, and are a major cause of morbidity and mortality [16,113]. Targeted TKIs have transformed the treatment paradigms for CNS involvement of NSCLC with oncogene-driven alterations, reforming them into upfront and effective therapies for CNS management. Next-generation TKIs achieve improved blood–brain barrier penetration, higher intracranial response rates, and longer PFS in comparison to conventional chemotherapy and earlier-generation TKIs [16,114]. For example, in ALK-positive NSCLC, lorlatinib demonstrated impressive CNS penetrance with an intracranial ORR of 82% and complete intracranial response rate of 71% in the CROWN trial [68]. Both ADC and bispecific antibodies have also shown intracranial response rates in advanced-stage NSCLC; however, the response rates are modest compared to next-generation TKIs [11,47].

Figure 2 summarizes a proposed framework for biomarker-guided sequencing and integration of TKIs, ADCs, and bispecific antibodies across the disease course of oncogene-driven NSCLC.

## 5. Toxicity Profiles and Quality-of-Life Considerations

Tyrosine kinase inhibitors (TKIs), antibody–drug conjugates (ADCs), and bispecific antibodies (bsAbs) each have distinct toxicity profiles that influence treatment selection, dose modifications, and patient quality of life.

### 5.1. Tyrosine Kinase Inhibitors Toxicities

EGFR-directed TKIs produce a well-characterized constellation of dermatologic and gastrointestinal adverse events. In the phase III FLAURA trial, osimertinib was associated with rash (58%), diarrhea (58%), and dry skin (36%), although grade ≥ 3 events were less frequent than with first-generation comparators (34% vs. 45%) [62]. QTc interval prolongation is a class-relevant concern: osimertinib demonstrates a concentration-dependent QTcF prolongation of approximately 16 msec at steady state, with 10% of patients experiencing prolonged QT interval as an adverse event [115]. A WHO pharmacovigilance analysis confirmed that osimertinib carries a significantly elevated reporting odds ratio for QT prolongation (ROR 6.13), heart failure (ROR 3.64), and supraventricular tachycardia relative to other EGFR TKIs [116]. ALK and ROS1 inhibitors as a class are associated with higher odds of conduction disease (ROR 12.95) and QT prolongation (ROR 5.16) compared with EGFR and BRAF inhibitors, with crizotinib carrying the highest individual risk [116]. FAERS network analysis further delineates class-specific signatures: ALK TKIs are enriched for metabolic and laboratory abnormalities (dyslipidemia with lorlatinib, creatine kinase elevation with brigatinib), while RET TKIs are associated with hepatotoxicity and hypertension [117]. These toxicities are generally manageable with oral administration, preserving patient autonomy and quality of life.

### 5.2. Antibody–Drug Conjugates Toxicities

Interstitial lung disease (ILD)/pneumonitis represents the most clinically consequential toxicity of ADCs, particularly those carrying topoisomerase I inhibitor payloads. A meta-analysis of trastuzumab deruxtecan (T-DXd) clinical trials reported an all-grade ILD/pneumonitis incidence of 12.5%, with grade ≥ 3 events in 2.2% [118]. A pooled analysis across nine DESTINY studies (n = 1678) confirmed adjudicated drug-related ILD in approximately 11–12% of patients, with the majority being grade 1–2 [119]. Notably, ILD incidence is highest in patients with non-small-cell lung cancer (22.2%) [48]. Mechanistic studies implicate off-target Fc-FcγR–mediated uptake of T-DXd by perivascular alveolar macrophages, triggering a pro-inflammatory SPP1-high phenotype [120]. In contrast, sacituzumab govitecan (TROP2-directed) does not exhibit a significant ILD signal in pharmacovigilance analyses, likely reflecting differences in payload (SN-38 vs. deruxtecan), linker chemistry, and drug/antibody ratio [121]. Beyond ILD, ADCs produce substantial gastrointestinal toxicity (nausea in 65–75%, vomiting) and hematologic effects (neutropenia in 35–49%), which frequently necessitate dose modifications [119]. These cumulative toxicities, combined with intravenous administration every three weeks, impose a meaningful burden on the patient’s quality of life.

### 5.3. Bispecific Antibodies Toxicities

Amivantamab, an EGFR-MET bispecific antibody, exemplifies the unique toxicity profile of this class. Infusion-related reactions (IRRs) are the hallmark of early toxicity, occurring in 63–67% of patients receiving intravenous formulations, predominantly during cycle 1 day 1 [12,122]. Most IRRs are grade 1–2 and rarely recur with subsequent infusions; prophylactic oral dexamethasone (8 mg twice daily) reduces IRR incidence approximately threefold [123]. Dermatologic toxicities driven by EGFR inhibition, such as acneiform rash (62%), paronychia (68%), and MET-mediated peripheral edema and hypoalbuminemia, are highly prevalent and, while generally low-grade, significantly affect quality of life [5] . In the MARIPOSA trial, grade ≥ 3 adverse events occurred in 75% of patients receiving amivantamab-lazertinib versus 43% with osimertinib, with skin/nail toxicity, venous thromboembolism (37%), and infusion reactions each requiring distinct prophylactic measures [5,12]. The subcutaneous formulation of amivantamab substantially reduces IRR rates (13% vs. 66%) and administration time (5 min vs. up to 5 h), representing a meaningful improvement in treatment experience [11]. Proactive dermatologic management with the COCOON regimen has also significantly reduced grade ≥ 2 cutaneous events [11].

## 6. Mechanisms of Resistance Across Modalities

Discussed below is the Mechanism of Resistance across all three classes of drugs. Table 2 explains this further. 

### 6.1. Tyrosine Kinase Inhibitors Mechanism of Resistance

Resistance to TKIs is broadly categorized into on-target (kinase domain) and off-target (bypass) mechanisms. For EGFR TKIs, the T790M gatekeeper mutation accounts for approximately 50–60% of resistance to first- and second-generation agents, while the C797S mutation is the predominant on-target mechanism following osimertinib [9,124]. Off-target bypass signaling, including MET amplification, HER2 amplification, KRAS mutations, BRAF fusions, and PIK3CA alterations, collectively accounts for a substantial proportion of post-osimertinib resistance [124,125]. Phenotypic transformation to small cell lung cancer or epithelial-mesenchymal transition represents an additional EGFR-independent escape mechanism [9,126]. For ALK-rearranged NSCLC, compound ALK kinase domain mutations (e.g., G1202R) confer resistance to second-generation inhibitors, while resistance to the third-generation agent lorlatinib involves compound mutations or bypass activation through similar pathways [9]. NCCN guidelines recommend molecular profiling at progression, including parallel circulating tumor DNA and tissue-based testing, to identify actionable resistance mechanisms and guide subsequent therapy [127].

### 6.2. Antibody–Drug Conjugates Mechanism of Resistance

ADC resistance is multifactorial, reflecting the tripartite structure of these agents. Target antigen loss or downregulation, demonstrated for HER2 in the DAISY trial and for TROP2 in preclinical models, reduces antibody binding and internalization [128,129]. Impaired intracellular trafficking, including defective endosomal-lysosomal processing and altered antibody recycling, limits payload release [130,131]. Upregulation of ATP-binding cassette (ABC) drug efflux transporters, particularly ABCG2 and ABCB1 (MDR1), directly reduces intracellular payload concentration; siRNA-mediated knockdown of these transporters restores T-DXd sensitivity in resistant models [132]. Payload-specific resistance through alterations in topoisomerase I (for deruxtecan-based ADCs) or tubulin dynamics (for DM1/MMAE payloads) further limits efficacy [129,131]. Strategies to overcome ADC resistance include switching to ADCs with alternative payloads (e.g., eribulin-based BB-1701 in T-DXd–resistant models), bispecific ADC designs, and combination with immune checkpoint inhibitors to leverage ADC-induced immunogenic cell death [132,133].

### 6.3. Bispecific Antibodies Mechanism of Resistance

Resistance to T-cell–engaging bispecific antibodies involves both tumor-intrinsic and immune-mediated mechanisms. Antigen escape through biallelic deletion, point mutations, or epigenetic silencing of target genes (e.g., BCMA, GPRC5D, CD19) is a well-documented tumor-intrinsic driver, with convergent evolution of antigen-negative subclones observed in multiple myeloma [134,135]. Loss of major histocompatibility complex class I expression impairs TCR co-stimulatory signaling required for optimal T-cell–mediated killing [135]. Tumor-extrinsic resistance is driven by T-cell exhaustion from chronic bispecific-mediated activation, characterized by upregulation of PD-1, TIGIT, and TIM-3, with reduced proliferative capacity and cytokine secretion [136]. The immunosuppressive tumor microenvironment—including regulatory T cells, myeloid-derived suppressor cells, and inhibitory cytokines—further impairs bispecific antibody efficacy [137,138]. For amivantamab specifically, resistance in solid tumors may involve EGFR or MET pathway alterations analogous to TKI resistance, though dedicated resistance profiling data remain limited. Combination strategies incorporating immunomodulatory agents, proteasome inhibitors (which upregulate death receptor expression), or sequential targeting of alternative antigens represent emerging approaches to overcome bispecific antibody resistance [135,137].

### 6.4. Cross-Resistance Considerations

When sequencing therapies across modalities, cross-resistance potential must be considered. TKI-induced bypass signaling (e.g., MET amplification) may reduce the efficacy of subsequent MET-targeting bispecifics. Conversely, antigen downregulation following ADC therapy could limit the activity of bispecific antibodies targeting the same antigen. Payload resistance mechanisms (e.g., topoisomerase I resistance) may cross-react with chemotherapy backbones used in combination regimens [124,133]. Molecular profiling at each progression point remains essential for rational therapy sequencing.

## 7. Future Directions

The future directions in lung cancer treatment include antibody–drug conjugates, trispecific antibodies and combination treatments. Antibody–drug conjugates (ADCs) are novel therapeutic targets in the treatment of lung cancer that targets tumor-specific antigens including TROP-2, HER-2, and HER-3. ADCs use linker-payload systems that enhance targeted treatment while reducing off-targeted side effects due to high selectivity [139]. Trispecific antibodies are considered the next-generation immunologic precision agents that can selectively target multiple tumor antigens and immune pathways (e.g., checkpoint modulation, dual T-cell activation) with increased specificity and increased antitumor activity compared to existing approaches [140]. Trispecific antibodies have been widely used in hematologic malignancies previously but are being investigated in solid malignancies such as lung cancer. Lung cancer management that encompasses combination treatments allows for synergistic treatment modalities with a decreased risk for resistance and increased response rate. For example, in EGFR-mutant NSCLC, combining a third-generation TKI such as lazertinib with a bispecific antibody such as amivantamab allows for a complementary antitumor activity by modulating extracellular receptor pathways and inhibiting intracellular kinase signaling with increased overall response in comparison to monotherapy with TKI alone [141]. Other potential treatment combinations still being investigated in early phase/clinical trials include TKI with ADCs and ADCs with bispecific antibodies, which may overcome resistance pathways, improve effectiveness, and improve patient outcomes in comparison to monotherapy alone.

Monitoring using longitudinal ctDNA allows for real-time assessment of the patient’s tumor status and treatment response earlier than other surveillance modalities, such as imaging. Detecting signs of progression or inadequate response to treatment may improve overall survival and progression-free survival in patients [142]. Longitudinal molecular profiling such as ctDNA and NGS testing allows for recognition of specific targets or resistance patterns which enables personalized and tailored treatments for patients with lung cancer [143]. Thus, it is important to use molecular profiling with NGS testing at the first sign of progression to guide the optimal treatment.

## 8. Conclusions

TKIs are currently the basis of first-line, targeted treatment for patients with NSCLC, especially in those that have actionable mutations such as EGFR, ALK, and others due to increased progression-free survival and overall survival [144]. ADCs and bispecific antibodies are changing the salvage and resistance-directed treatments enabling targeted delivery and dual inhibition of pathways after progression on other treatment modalities. Targeted agents such as TROP-2- and HER-3-directed ADCs and MET bispecific antibodies allow for increased response rates in heavily treated patients, especially in those with resistance [145,146,147]. Based on emerging evidence, these modalities will coexist while offering distinct advantages. In the future, the optimal treatment will likely involve combination treatments using all three drug class lines after utilizing precision-guided treatment using molecular biomarkers to help guide treatment sequencing.

## Figures and Tables

**Table 1:** Comparative Molecular Characteristics of TKIs, ADCs, and Bispecific Antibodies.

Characteristic	TKIs	ADCs	Bispecific Antibodies (bsAbs)
Molecular size	Small (~300–600 Da)	Large (~150 kDa)	Large (~150 kDa)
Route of administration	Oral	Intravenous	Intravenous (subcutaneous in development)
Primary mechanism	ATP-competitive or allosteric kinase inhibition	Antibody-mediated targeting + cytotoxic payload delivery	Dual receptor blockade + immune effector engagement (ADCC, ADCT)
Bystander effect	None	Yes (cleavable linkers with membrane-permeable payloads)	Limited
Dependence on target expression	Requires activating kinase mutation	Requires surface antigen expression	Requires surface receptor expression
CNS penetration	High (third-generation: osimertinib, lorlatinib)	Limited; emerging intracranial activity via BBB disruption	Limited
Half-life	Hours (6–48 h)	Days (5–7 days)	Days (5–7 days)
Key resistance mechanisms	On-target mutations (C797S, G1202R), bypass signaling (MET, HER2/3)	Antigen loss, drug efflux pumps, payload resistance	Receptor downregulation, decreased FcγR engagement
Key advantages	Molecular precision, oral convenience, excellent CNS activity	Payload-driven potency independent of resistance mutations, bystander killing	Dual pathway blockade, immune engagement, overcomes bypass resistance
Key limitations	Susceptible to point mutations and bypass activation	Large size limits CNS penetration; ILD/pneumonitis risk	IV administration, infusion reactions, limited CNS activity

**Table 2:** Resistance Mechanisms Across Therapeutic Classes.

Mechanism Category	TKIs	ADCs	Bispecific Antibodies (bsAbs)	References
On-Target Alterations	Secondary kinase mutations (e.g., EGFR C797S, ALK G1202R, L718Q, L792H)	Antigen loss or downregulation (e.g., HER2, TROP2, c-MET)	Receptor downregulation via internalization and degradation	[8,9,10,11,45,46,47,48]
Bypass Pathway Activation	MET amplification, HER2/HER3 amplification, RAS/MAPK pathway activation	Less dependent on bypass signaling due to payload-mediated cytotoxicity	Reduced efficacy when resistance is driven by pathways not targeted by the bispecific construct	[8,11,16,45,46,47,48]
Downstream Signaling Alterations	PI3K/AKT pathway activation, cell-cycle gene alterations (RB1, CDKN2A), MAPK reactivation	Payload-specific resistance mechanisms (e.g., tubulin alterations, topoisomerase I resistance)	Reactivation of downstream signaling pathways despite receptor blockade	[8,16,19,20,26,28]
Histologic Transformation	Small cell lung cancer transformation (~5–10%), squamous transformation	May retain efficacy if target antigen expression is preserved	Clinical efficacy after histologic transformation remains uncertain	[8,16]
Drug Efflux and Metabolism	Not a dominant mechanism	Increased expression of drug efflux pumps (e.g., P-glycoprotein, BCRP), altered payload metabolism	Not a major resistance mechanism	[23,32,33,34]
Immune Evasion	Not a primary mechanism	Reduced immunogenic cell death and altered tumor microenvironment	Decreased Fc-mediated immune engagement, impaired ADCC/ADCT activity, immunosuppressive tumor microenvironment	[49,50,51]
Polyclonal Resistance and Tumor Heterogeneity	Multiple concurrent resistance mechanisms frequently coexist, particularly after osimertinib	Heterogeneous antigen expression may reduce uniform payload delivery	Multiple receptor alterations and signaling redundancy may limit efficacy	[10,11,29,30]

Abbreviations: ADC, antibody–drug conjugate; ADCC, antibody-dependent cellular cytotoxicity; ADCT, antibody-dependent cellular trogocytosis; BCRP, breast cancer resistance protein; bsAb, bispecific antibody; HER2, human epidermal growth factor receptor 2; MAPK, mitogen-activated protein kinase; MET, mesenchymal–epithelial transition receptor; PI3K, phosphoinositide 3-kinase; TKI, tyrosine kinase inhibitor.

## Data Availability

No new data were created or analyzed in this study. Data sharing is not applicable to this article.

## Abbreviations

The following abbreviations are used in this manuscript:

ABC	ATP-Binding Cassette (transporter family)
ABCB1	ATP-Binding Cassette Subfamily B Member 1 (MDR1/P-glycoprotein)
ABCG2	ATP-Binding Cassette Subfamily G Member 2 (BCRP)
ADC	Antibody–Drug Conjugate
ADCC	Antibody-Dependent Cellular Cytotoxicity
ADCP	Antibody-Dependent Cellular Phagocytosis
ADCT	Antibody-Dependent Cellular Trogocytosis
ALK	Anaplastic Lymphoma Kinase
ASCO	American Society of Clinical Oncology
ATP	Adenosine Triphosphate
B7-H3	B7 Homolog 3 (CD276)
BBB	Blood–Brain Barrier
bsAb	Bispecific Antibody
BCRP	Breast Cancer Resistance Protein
c-MET	Mesenchymal–Epithelial Transition Receptor
CNS	Central Nervous System
ctDNA	Circulating Tumor DNA
CTLA-4	Cytotoxic T-Lymphocyte Associated Protein 4
DAR	Drug-to-Antibody Ratio
DXd	Deruxtecan Payload
EGFR	Epidermal Growth Factor Receptor
ERBB2	Erb-B2 Receptor Tyrosine Kinase 2 (HER2)
HER2	Human Epidermal Growth Factor Receptor 2
HER3	Human Epidermal Growth Factor Receptor 3
ICIs	Immune Checkpoint Inhibitors
ILD	Interstitial Lung Disease
LAG-3	Lymphocyte Activation Gene 3
MAPK	Mitogen-Activated Protein Kinase
METex14	MET Exon 14 Skipping Mutation
MGPT	Multigene Panel Testing
MMAE	Monomethyl Auristatin E
NCCN	National Comprehensive Cancer Network
NGS	Next-Generation Sequencing
NRG1	Neuregulin 1
NSCLC	Non-Small-Cell Lung Cancer
ORR	Objective Response Rate
OS	Overall Survival
PD-1	Programmed Cell Death Protein 1
PD-L1	Programmed Death-Ligand 1
PFS	Progression-Free Survival
PI3K	Phosphoinositide 3-Kinase
RET	Rearranged During Transfection
ROR	Reporting Odds Ratio
ROS1	ROS Proto-Oncogene 1
TIM-3	T-cell Immunoglobulin and Mucin Domain-containing Protein 3
TIGIT	T-cell Immunoreceptor with Ig and ITIM Domains
TMB	Tumor Mutational Burden
T-DM1	Ado-Trastuzumab Emtansine
T-DXd	Trastuzumab Deruxtecan
TKI	Tyrosine Kinase Inhibitor
TME	Tumor Microenvironment
TROP2	Trophoblast Cell Surface Antigen 2
VEGFR	Vascular Endothelial Growth Factor Receptor
VISTA	V-domain Ig Suppressor of T-cell Activation
VTE	Venous Thromboembolism
